# The Effect of the Maxillary Sinus Volume on the Morphology and Angulation of the Infraorbital Canal in Relation to Age and Gender

**DOI:** 10.1007/s00266-025-04719-w

**Published:** 2025-02-12

**Authors:** Gülay Açar, Ahmet Safa Gökşan, Guldane Magat

**Affiliations:** 1 Department of Anatomy, Faculty of Medicine, Necmettin Erbakan University, 42090 Meram, Konya, Turkey; 2 Department of Anatomy, Faculty of Medicine, Aksaray University, 68100 Merkez, Aksaray, Turkey; 3 Department of Oral and Maxillofacial Radiology, Faculty of Dentistry, Necmettin Erbakan University, 42090 Meram, Konya, Turkey

**Keywords:** Infraorbital canal types, Maxillary sinus pneumatization, Transmaxillary procedures, Three-dimensional volumetric analysis, Cone beam computed tomography

## Abstract

**Background:**

Although the infraorbital canal (IOC) and maxillary sinus (MS) have been well studied, understanding the effect of MS volume (MSV) on IOC morphology is critical in determining the safest surgical route for infraorbital depression and transmaxillary procedures.

**Objectives:**

We aimed to describe the IOC types, measure the MSV and IOC angles (IOCA) in all three planes, and analyse the relationship between them using three-dimensional (3D) cone beam computed tomography (CBCT) images.

**Methods:**

CBCT images of 280 patients were analysed to identify the IOC types and accessory IOC (IOCa), and to measure morphometric parameters. The relationship between them was examined using statistical analysis in relation to age, gender, and laterality.

**Results:**

The most common IOC type was Type I (59.6%), followed by Type II (21.8%), Type III (13.6%), and Type IV (5%). According to MSV, three types of MS were described, with 38.2, 34.6, and 27.2% having normal, hypoplastic, and hyperplastic MS, respectively. Also, hyperplastic MS was associated with the highest likelihood of Type III IOC. Furthermore, logistic regression model revealed that the MSV and IOCA3 had a positive significant effect on the IOC protrusion, whereas being female, increasing age and IOCA1 had a negative significant effect on MS pneumatization. The probability of having hyperplastic MS, Types II and III IOC, IOCa also decreased with increasing age.

**Conclusions:**

Using 3D technology, the results of this study provide a detailed classification of IOC and MS types, increasing the number of treatment options and reducing the risk of complications during surgery.

**Level of Evidence IV:**

This journal requires that authors assign a level of evidence to each article. For a full description of these Evidence-Based Medicine ratings, please refer to the Table of Contents or the online Instructions to Authors www.springer.com/00266.

## Introduction

Knowledge of the variable morphology and morphometric characteristics of the infraorbital canal (IOC) is essential for progress in planning of various midface, orbital, and periocular procedures including filler injections, fat grafting, lower blepharoplasty, laceration repair, and surgical repair of midface or blow-out fractures [1, 2]. These data significantly improve the outcome of diagnostic regional block anaesthesia and lower eyelid surgery [2–4]. As already known, the IOC is the continuation of the infraorbital groove in the maxillary sinus (MS) roof, through which the infraorbital nerve (ION) and artery (IOA) pass. Its facial opening (infraorbital foramen, IOF) is located below the inferior orbital rim. The ION and IOA provide sensory innervation and vascular supply to the malar region between the lower eyelid region and the upper lip, respectively [1–7]. The MS is the largest and deepest of the paranasal sinuses, which can vary in size and shape. The roof of the MS forms the orbital floor, and the base of the MS can extend down to the tooth roots [8–10].

More recently, a detailed three-dimensional (3D) assessment of MS and IOC can be easily performed using cone beam computed tomography (CBCT) scans, which are low-dose, accessible, and easy-to-use. Volume rendering is becoming increasingly popular for preoperative assessment, diagnosis, and surgical treatment planning [3, 4, 7–10].

Although many studies have been published on IOC and MS morphology individually, there are few studies demonstrating differences in IOC shape, location, and morphometry depending on MS pneumatization (MSP) [2–7]. Variable IOC protrusion into the MS influences the risk of neurovascular injury if not recognized during surgery, while reduced MSP may result in lateral displacement of the IOF and inferomedial positioning of the orbital floor [2, 11–17]. MS hypoplasia was commonly associated with mucosal thickening, which compromised vascularization and decreased implant success [12]. Several studies have reported that high IOC protrusion, more common with MS hyperplasia, is associated with longer septum indicating an increased risk of traumatic injury [11, 12, 14, 15, 17].

However, no consensus has been reached on how MSP affects IOC morphology and IOF location as related to age and gender. Therefore, we aimed to assess the MSP, the frequency of IOC protrusion into the MS, and to identify other IOC variants and angles that may increase the likelihood of iatrogenic injury. Using 3D CBCT images, we also attempted to determine the co-existence of these variables and the influence of age and gender. To avoid misinterpretation during surgical or cosmetic procedures in the midface and orbital floor region, it is important to know how IOC morphology relates to different MSP types and how age and gender affect this.

## Materials and Methods

This study was approved by the University’s Research Ethics Committee with an approval number 2023/4455. A total of 280 CBCT scans were retrieved from the archives of the Department of Oral and Maxillofacial Radiology between January 2023 and May 2024. CBCT images were acquired using a 3D Accuitomo 170 scanner (J. Morita Corp., Kyoto, Japan). The operating parameters were set to 90 kV, 5 mA, and a scan time of 17.5 s (scan dimensions of 17 × 12 cm). All CBCT images were converted into DICOM (Digital Imaging and Communications in Medicine) files for further analysis. Three-dimensional Slicer (open-source software platform) was used to create the 3D models in this study. All measurements were made by an experienced radiologist to prevent inter-observer variability.

Exclusion criteria included images from individuals with a history of maxillofacial trauma or surgery, MS pathology, congenital anomaly (cleft lip or palate), any missing teeth in the upper jaw (except the third molars), dental implants or spaced dentition, poor quality, or artefact images that would make evaluation of the MS and IOC impossible. Patients without MS pathology aged ≥18 years were included.

**Fig. 1:**
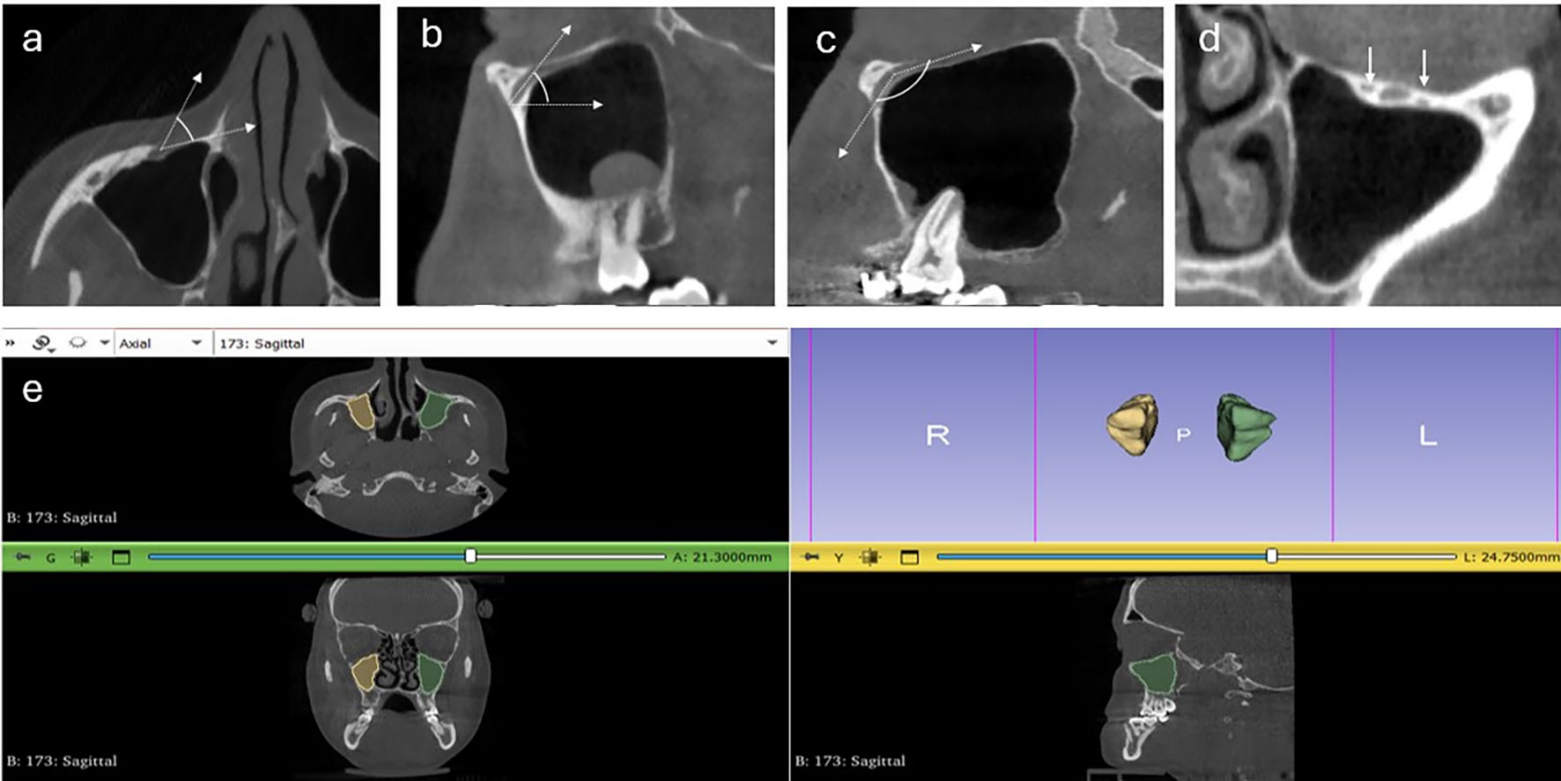
**a** Axial CBCT image showing infraorbital canal angle (IOCA)1 constructed between the IOC and the horizontal axis, **b** sagittal CBCT image showing IOCA2 constructed between the IOC and the sagittal axis, **c** sagittal CBCT image showing IOCA3 constructed between the IOC and infraorbital groove, **d** coronal CBCT image showing two accessory IOCs, and **e** three-dimensional reconstruction of maxillary sinus (MS) and volume measurement

The following morphometric parameters:

IOCA1: The angle constructed between the IOC axis and the horizontal axis that parallel to the nasal floor with the vertex at the centre of the IOF in the axial plane (Fig. 1a).

IOCA2: The angle constructed between the IOC axis and the sagittal axis in the sagittal plane (Fig. 1b).

IOCA3: The angle constructed between the IOC and IOG in the sagittal plane (Fig. 1c).

**Fig. 2:**
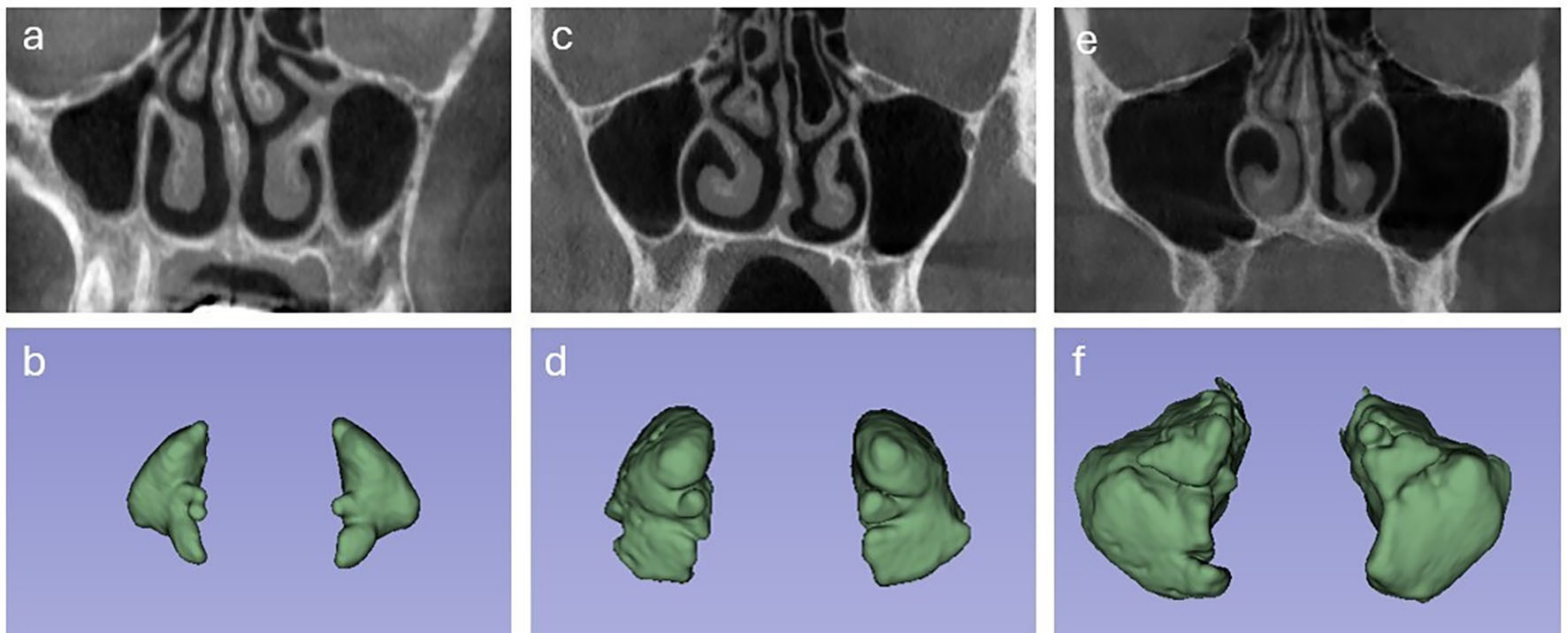
Coronal CBCT image and three-dimensional reconstruction of, **a-b** hypoplastic maxillary sinus (MS), **c-d** normal MS, and **e-f** hyperplastic MS

**Fig. 3:**
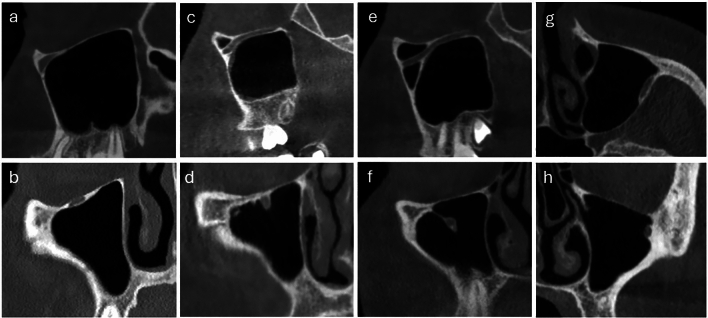
Sagittal and coronal CBCT images of, **a-b** Type I infraorbital canal (IOC), **c-d** Type II IOC, **e-f** Type III IOC on the right side, **g-h** axial and coronal CBCT image of Type IV IOC on the left side, and **h** IOC dehiscence

The number of accessory IOC (IOCa) was also noted (Fig. 1d). The boundaries of the MS were drawn separately in each section. All sections were automatically combined using 3D Slicer software, and MSV was calculated (Fig. 1e) and the MSP types were classified into three groups according to MSV:

Hypoplastic MS: The MSV was between 1.4 and 11.99 cm^3^ (Fig. 2a and b),

Normal MS: The MSV was between 12 and 19.99 cm^3^ (Fig. 2c and d),

Hyperplastic MS: The MSV was between 20 and 34 cm^3^ (Fig. 2e and f),

Four types of IOC were identified according to the degree of IOC protrusion into the MS, as follows:

Type I: The IOC is located entirely within the roof of the MS (Fig. 3a and b),

Type II: The IOC is located under the roof of the MS with partial protrusion (Fig. 3c and d),

Type III: The IOC protruded completely into the MS or suspended from the roof of the MS by a septum or lamella (Fig. 3e and f),

Type IV: The IOC showed a lateroantral course within the roof of the MS. It was located at the outer border of the zygomatic recess. Also, IOC dehissence (IOCd) was noted (Fig. 3g and h).

## Statistical Analysis

SPSS version 25.0 (IBM SPSS Statistics, Armonk, NY, USA) was used for data analysis. The morphometric parameters were subjected to the Shapiro–Wilk normality test, while differences between categorical variables were analysed using Kruskal–Wallis test. All measurements were compared between the right and left sides using a paired sample t-test, while a Student's t-test was used to detect significant gender differences. The frequency distribution of categorical variables was analysed using the Chi-square test (χ2 test). Analysis of variance (ANOVA) test allowed the assessment of the variance between dependent and independent variables. Logistic regression analysis was performed to assign predictive variables for IOC types and MSP types. Intraclass correlation coefficient (ICC) was used to assess the intra-observer reliability. The significance level was set at *p* < 0.05.

## Results

Of the 280 patients (range 18–78 years, mean age 37.31 ± 14.27 years) in this retrospective study, 143 were female (mean age 38.66 ± 14.95 years) and 137 were male (mean age 35.89 ± 13.7 years). The mean IOCA1, IOCA2, and IOCA3 in our study were measured as 36.45±9.5°, 49.35±8.0°, and 143.44±8.7°, respectively. The mean MSV that was divided into three types was 15.20±6.7 cm^3^ and was greater in males than females (*p* = 0.000). The most common type of MSP was normal MS (56 females and 51 males, 38.2%), followed by hypoplastic MS (55 females and 42 males, 34.6%) and hyperplastic MS (32 females and 44 males, 27.2%). As shown in Table 1, the prevalence of hypoplastic MS was higher in females, while the prevalence of hyperplastic MS was higher in males with a significant gender difference (*p* = 0.025, χ2=7.354). The mean IOCA1 was larger in males than in females (*p* = 0.000), whereas females presented a wider IOCA2 and IOCA3 than males, although no significant differences were found (*p* = 0.184 and *p* = 0.905, respectively). With regard to laterality, the mean MSV was greater on the right side than on the left (*p* = 0.046), whereas the mean value of IOCA1, IOCA2, and IOCA3 were side independent (*p* = 0.651, *p* = 0.105, and *p* = 0.301, respectively) (Table 2).

**Table 1:** Comparison of sample variants and morphometric measurements according to gender

		Female (*n* = 143)	Male (*n* = 137)	*p* value
Maxillary sinus pneumatization types	Hypoplastic	110 (38.5%)	84 (31%)	
	Normal	112 (39.5%)	102 (37.2%)	p= 0.025*
	Hyperplastic	64 (22%)	88 (31.8%)	χ^2^= 7.354
Infraorbital canal types	Type I	184 (62.6%)	150 (56.9%)	
	Type II	58 (20.3%)	64 (23%)	p = 0.150
	Type III	36 (14%)	40 (13.1%)	χ^2^ = 5.313
	Type IV	8 (3.1%)	20 (6.9%)	
Infraorbital canal dehiscence	Absent	226 (79%)	192 (70.1%)	p= 0.027*
	Present	60 (21%)	82 (29.9%)	χ^2^ = 7.157
Accessory infraorbital canal	Absent	248 (86.7%)	216 (79.2%)	p = 0.039*
	1	34 (12.2%)	56 (20.1%)	χ^2^ = 6.457
	2	4 (1%)	2 (0.7%)	

Morphometric data	Female Mean ± SD	Male Mean ± SD	*p* value
Age	38.66±16.5	35.89±13.7	0.032*
IOCA1 (^o^)	35.66±8.9	37.27±9.8	0.044*
IOCA2 (^o^)	49.81±8.1	48.91±7.9	0.184
IOCA3 (^o^)	143.48±8.2	143.39±9.2	0.905
MSV (cm^3^)	14.12±6.1	16.32±7.2	0.000*

* Statistically significant p value, χ2 Pearson Chi-square test result, Student's *t*-test result, *IOCA* infraorbital canal angle, and *MSV* maxillary sinus volume

**Table 2:** Comparison of the sample variants and morphometric measurements according to laterality

		Right	Left	Bilateral	*p* value
		n (%)	n (%)	n (%)	
Maxillary sinus pneumatization types	Hypoplastic	12 (11%)	12 (11%)	85 (78%)	0.000*
	Normal	20 (15.7%)	20 (15.7%)	87 (68.6%)	
	Hyperplastic	8 (9.4%)	10 (11.8%)	67 (78.8%)	
Infraorbital canal types	Type I	12 (6.4%)	26 (14%)	148 (79.6%)	0.000*
	Type II	26 (31%)	20 (23.8%)	38 (45.2%)	
	Type III	16 (30.8%)	12 (23.1%)	24 (46.1%)	
	Type IV	2 (14.3%)	2 (14.3%)	12 (71.4%)	
Infraorbital canal dehiscence	Absent	20 (8.7%)	24 (10.4%)	186 (80.9%)	0.000*
	Present	22 (23.9%)	20 (21.7%)	50 (54.4%)	
Accessory infraorbital canal	Absent	10 (4%)	26 (10.4%)	214 (85.6%)	0.000*
	1	20 (33.3%)	10 (16.7%)	30 (50%)	
	2	4 (66.7%)	2 (33.3%)	0 (0%)	

Morphometric data	Right Mean±SD	Left Mean±SD	*p* value
IOCA1 (^o^)	36.34±9.8	36.55±9.1	0.651
IOCA2 (^o^)	49.20±7.8	48.49±6.9	0.105
IOCA3 (^o^)	143.60±9.6	143.28±7.7	0.301
MSV (cm^3^)	15.36±6.9	15.03±6.5	0.046*

* Statistically significant * p* value, paired* t*-test result, *IOCA* infraorbital canal angle, and *MSV* maxillary sinus volume

In terms of IOC types, Type I was the most common (59.6%), followed by Type II (21.8%), Type III (13.6%), and Type IV (5%). There was no significant gender difference in the frequency of them (*p* = 0.150, χ2=5.313), whereas the frequencies of IOCd and IOCa were higher in males than in females (*p* = 0.039, χ2=6.457; *p* = 0.027, χ2=7.157, respectively) (Table 1). Furthermore, IOC types were more likely to be bilateral (*p* = 0.000), as were MSP types (Table 2).

**Fig. 4:**
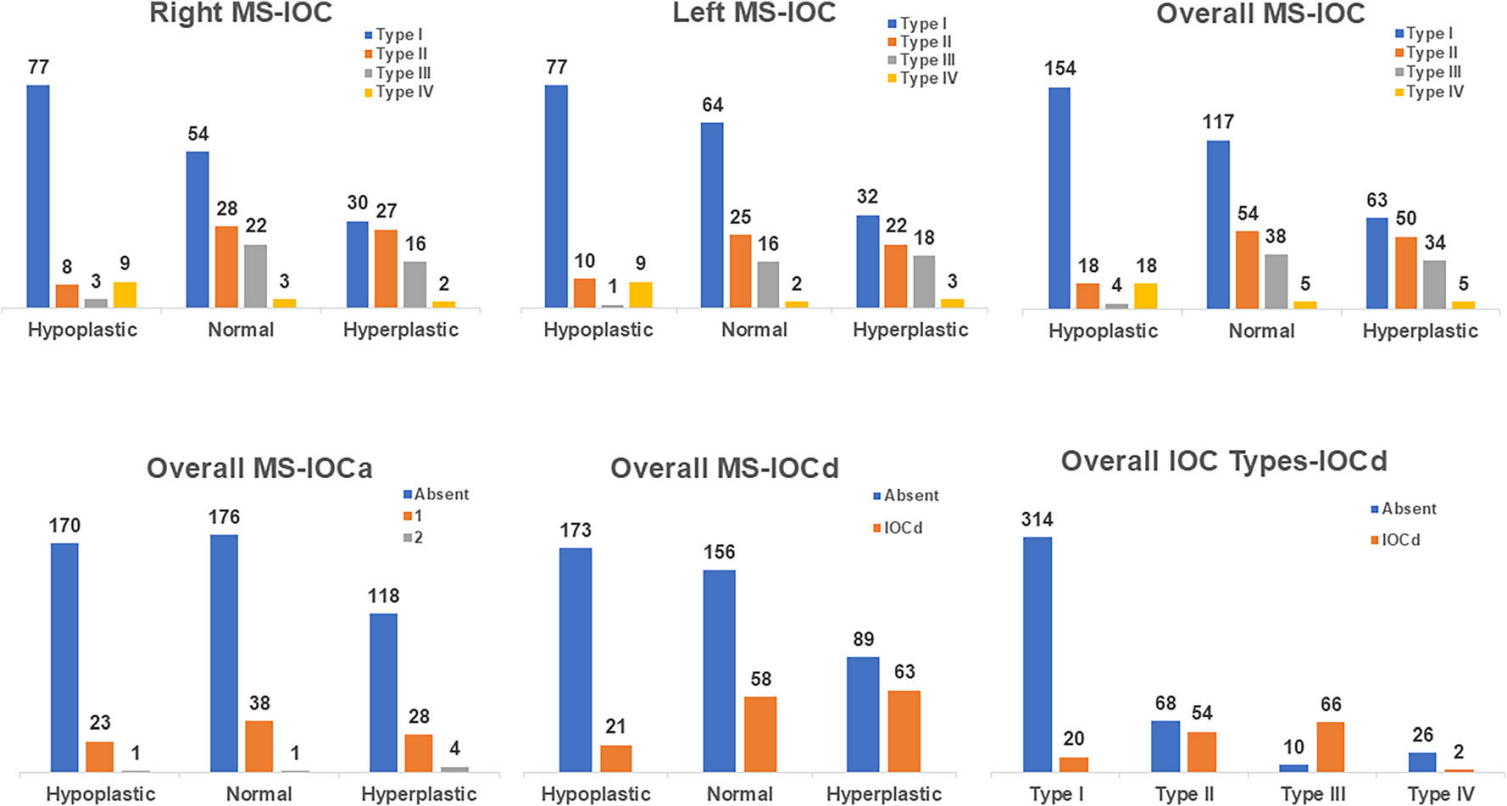
Bar diagrams showing the relationship between the maxillary sinus pneumatization (MSP) types, infraorbital canal (IOC) types, accessory IOC, and IOC dehiscence on the right–left sides and overall

**Fig. 5:**
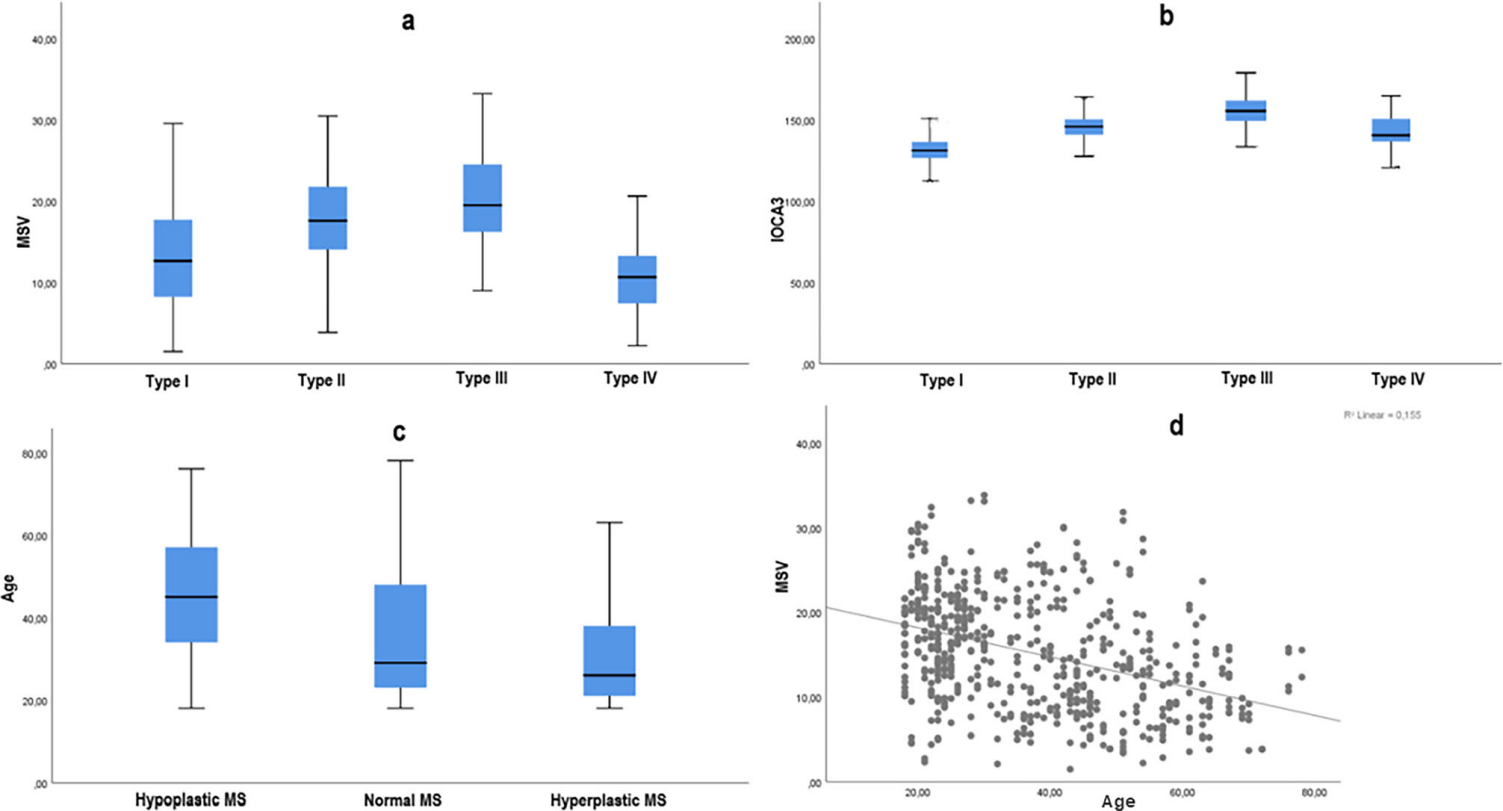
Scatter plots showing the relationship between **a** the types of infraorbital canal (IOC) and maxillary sinus volume (MSV), **b** the types of IOC and infraorbital canal angle (IOCA)3, **c** the maxillary sinus pneumatization (MSP) types and age, and **d** the MSV and age

Type III IOC was found to be significantly more prevalent in hyperplastic MS, whereas hypoplastic MS was associated with a higher frequency of Types I and IV IOCs (*p* = 0.000). Increased MSV also has a positive effect on IOC protrusion and dehiscence rates. The incidence of IOCd was significantly higher in patients with Types II and III. The detailed information regarding the number of each IOC, IOCa, and IOCd in the MSP types (right, left, and overall) are given in Fig. 4, and the statistical association between them can be seen in Table 3. In Table 4, there appears to be a similar general tendency for mean IOCA3 and MSV to be greater in Types II and III IOCs (Fig. 5a and b), whereas hypoplastic MS was associated with increased age and IOCA1 (*p* = 0.000).

**Table 3:** The relationship between maxillary sinus pneumatization types and infraorbital canal variations

Relationship	*p* value	df	CC
RIOC and RMSP types	0.000*	6	0.381
RIOCa and RMSP types	0.512	4	0.108
LIOC and LMSP types	0.000*	6	0.360
LIOCa and LMSP types	0.118	4	0.161
OIOC and OMSP types	0.000*	6	0.367
OIOCa and OMSP types	0.123	4	0.113
OIOCd and OMSP types	0.001*	3	0.287
OIOCd and OIOC types	0.000*	3	0.372

* *p* value shows statistically significant Chi-square test result, *df* degree of freedom, *CC* contingency coefficient degree of association, *IOC* infraorbital canal, *MSP* maxillary sinus pneumatization, *IOCa* accessory infraorbital canal, IOCd infraorbital canal dehiscence, *R* right, L left, and O overall.

**Table 4:** Comparison of the morphometric measurements according to IOC and MSP types

	Type I Mean ± SD	Type II Mean ± SD	Type III Mean ± SD	Type IV Mean ± SD	*p* value
Age	39.14±15.2	33.88±14.6	35.04±16.5	39.39±15.7	0,065
IOCA1 (^o^)	36.84±8.8	35.71±10.0	34.84±10.4	36.41±8.6	0,101
IOCA2 (^o^)	49.88±7.9	49.08±7.7	49.04±8.6	49.05±8.9	0,220
IOCA3 (^o^)	142.32±8.3	145.28±9.5	145.68±7.9	142.25±12.0	0,001*
MSV (cm^3^)	13.47±6.4	17.66±5.9	20.27±5.6	11.46±5.6	0,000*

MSP types	Hypoplastic MS Mean ± SD	Normal MS Mean ± SD	Hyperplastic MS Mean ± SD	*p* value
Age	44.73±14.9	35.76±15.4	29.89±10.7	0,000*
IOCA1 (^o^)	37.67±8.8	37.16±9.5	33.85±9.9	0,000*
IOCA2 (^o^)	48.76±8.4	49.32±7.9	50.18±7.6	0,263
IOCA3 (^o^)	142.60±9.2	143.47±9.4	144.20±7.6	0,098

* Statistically significant p value shows the results of ANOVA test, IOCA infraorbital canal angle, MSV maxillary sinus volume, and MSP maxillary sinus pneumatization

Ordinal logistic regression analyses were used to show the main effects of gender, age, and IOCAs on MSV and IOC types, as shown in Tables 5 and 6. In particular, increases in both age and IOCA1 and, to a lesser extent, gender (being female) have a significant decreasing effect on MSV. The overall model was significant (Wald=108.203; *p* = 0.000), indicating that age (Wald=52.938; *p* = 0.000), IOCA1 (Wald=16.036; *p* = 0.000), and gender (Wald=4.363; *p* = 0.037) have a significant effect on MSV. In Table 6, both MSV and IOCA3 retained a significant effect in the model (Wald=69.052, *p* = 0.000 and Wald=11.024, *p* = 0.001, respectively), in which Types II and III IOCs were more likely to be associated with larger MS and IOCA3, whereas gender and age had no significant effect.

**Table 5:** Ordinal logistic regression analysis for maxillary sinus pneumatization types

Variables	Estimate	SE	Wald	*p* value	95% CI
Model fit	–	–	108.203	0.000*	–
Gender	–0.341	0.163	4.363	0.037*	–0.660 to –0.021
Age	–0.055	0.012	52.938	0.000*	–0.078 to –0.031
IOCA1	–0.035	0.009	16.036	0.000*	–0.052 to –0.018
IOCA2	0.018	0.011	2.936	0.087	–0.003–0.040
IOCA3	0.007	0.010	0.440	0.507	–0.013–0.026

* *p* value shows statistically significant main effect result, *SE* standard error, *Wald* Chi-square *CI* confidence interval, and IOCA infraorbital canal angle

**Table 6:** Ordinal logistic regression analysis for infraorbital canal types

Variables	Estimate	SE	Wald	*p* value	95% CI
Model fit	–	–	110.206	0.000*	–
Gender	0.355	0.193	3.374	0.066	–0.024–0.733
Age	0.007	0.007	1.029	0.310	–0.006–0.020
MSV	0.141	0.017	69.052	0.000*	0.108–0.174
IOCA1	–0.002	0.010	0.045	0.833	–0.022–0.017
IOCA2	–0.006	0.012	0.253	0.615	–0.030–0.018
IOCA3	0.038	0.012	11.024	0.001*	0.016–0.061

* *p* value shows statistically significant main effect result, *SE* standard error, *Wald* Chi-square *CI* confidence interval, MSV maxillary sinus volume, and IOCA infraorbital canal angle

Three groups were created according to ages respectively as Group I (18–25 years) with 90 (32.1%) patients, Group II (26–44 years) with 100 (35.8%) patients, and Group III (45–78 years) with 90 (32.1%) patients. Table 7 shows that the smaller the MSV, the lower the IOC protrusion, and the greater the IOCA1 in older patients (*p* = 0.000). The degree of IOCA3 and MSV decreased significantly with increasing age (Fig. 5c); in other words, increasing age leads to hypoplastic MS (Fig. 5d). Overall, the ICC values were found between the range of 0.865 and 0.976.

**Table 7:** Comparison of sample variants and morphometric measurements according to age groups

Morphological data		Group I (18–25 years)	Group II (26–44 years)	Group III (45–78 years)	*p* value
Maxillary sinus pneumatization types	Hypoplastic	31 (17.2%)	64 (32%)	100 (55.6%)	0.000*
	Normal	82 (45.6%)	69 (34.5%)	64 (35.6%)	
	Hyperplastic	67 (37.2%)	67 (33.5%)	16 (8.9%)	
Infraorbital canal types	Type I	91 (50.6%)	120 (60.0%)	124 (68.9%)	0.011*
	Type II	50 (27.8%)	41 (20.5%)	30 (16.7%)	
	Type III	30 (16.7%)	25 (12.5%)	21 (11.7%)	
	Type IV	9 (5.0%)	14 (7.0%)	5 (2.8%)	
Accessory infraorbital canal	Absent	142 (78.9%)	172 (86.0%)	151 (83.9%)	0.124
	1	35 (19.4%)	27 (13.5%)	28 (15.6%)	
	2	3 (1.7%)	1 (0.5%)	1 (0.6%)	
Infraorbital canal dehiscence	Absent	118 (65.6%)	142 (71%)	158 (87.8%)	0.035*
	Present	62 (34.4%)	58 (29%)	22 (12.2%)	

Morphometric data	Group I Mean ± SD	Group II Mean ± SD	Group III Mean ± SD	*p* value
IOCA1 (^o^)	34.5±8.5	35.5±10.5	38.4±9.1	0.000*
IOCA2 (^o^)	47.7±8.8	48.7±7.6	46.4±7.2	0.108
IOCA3 (^o^)	145.4±8.8	143.1±8.0	141.9±8.9	0.000*
MSV (cm^3^)	17.56±6.2	16.16±6.7	11.77±6.0	0.000*

* Statistically significant p value shows the results of ANOVA test, *IOCA* infraorbital canal angle, and *MSV* maxillary sinus volume.

## Discussion

Many factors affecting the IOC morphology have been mentioned in the previous studies, including gender, age, laterality, different skeletal growth patterns, and surgical interventions. A review of the literature revealed a limited number of publications addressing the association between IOC types, IOCAs, IOCd, IOCa, and MSV from an anatomical perspective. This study demonstrated that Types II and III IOCs, mostly including IOCd, were significantly more common in patients with hyperplastic MS, whereas hypoplastic MS was associated with Types I and IV IOCs.

The anatomical relationship of IOC types to the MSP may influence the identification of the IOF as a landmark for anaesthetic applications. Accurate identification of IOF and puncture direction is essential to ensure safety and efficacy of the ION block. Depending on the IOCAs, the clinician inserted the needle into the IOF and advanced it upward and laterally along the IOC to perform regional block anaesthesia. It is important to be aware of the length and angulation of the IOC to avoid damage to orbital structures [2, 6]. In the previous studies, the mean values of the IOCA1, IOCA2, and IOCA3 were reported in a wide range between 13° and 36°, 46° and 58°, and 117° and 146°, which is consistent with the present study [3–7]. In our study, the mean value of the IOCA1 was larger in Types I and IV IOCs, while IOCA3 was larger in Types II and III IOCs with a significant difference (*p* = 0.001).

Regarding the IOC types, Type I was the most common (59.6%), followed by Type II (21.8%), Type III (13.6%), and Type IV (5%) in our study. Similarly, the highest frequency of Type I was reported by the previous studies ranging from 46.7 to 78.1% [2, 3, 8, 11, 14–16]. Only Li et al. [17] reported a relatively high Type II rate of 60%. They reported that Type I IOC (not bulge into the MS) and Type III IOC (orbital floor above the IOC) allow a direct view of the entire roof of the MS through endoscopic prelacrimal approach, whereas Type II IOC (42%) prevents visualization of lateral orbital floor and results in the need for an angled endoscope or transposition of the ION. In older patients, the orbital floor is also less rigid, leading to orbital floor fractures that can easily extend into the IOC or laterally. Moreover, they observed that Types II and III IOCs are most commonly associated with IOCd (40%), which may provide pathways for the spread of MS pathology to the ION [17]. In our study, the IOCd was most common in Types II and III IOCs in accordance with this study, but the prevalence was lower at 25.4%. The high incidence of IOCd may be related to increased contact with inflamed tissue or tumour lesions leading to intractable neuropathic pain or trigeminal neuralgia. Type III rates, the least common reported in the literature, vary between 7.9 and 14.6%. Ference et al. [16] highlighted that the longer the septum, the more the IOC extends into the sinus, indicating an increased risk of injury during a Caldwell-Luc approach, surgical repair of blow-out fractures, and Le Fort-type osteotomies. Serindere et al. [14] advised surgeons to consider septa attached to the sinus roof, especially in Type III IOC, when performing functional endoscopic sinus surgery. Type IV IOC (range 3–8.5%), in which the IOF is located laterally at the outer border of the zygomatic recess and its lateroantral course is almost parallel to the orbital floor, may alter the puncture directions for the ION block [2, 6, 18]. Lateral displacement of the IOF may result in poor anaesthetic efficacy or iatrogenic ION injury.

Recently, the widespread use of 3D modelling has been shown to provide a detailed information about the IOC morphology, as well as 3D model-based volumetric measurement of the MS [8–12, 15, 18, 19]. We found that the mean MSV was 15.20±6.7 cm^3^, which is consistent with the results of the previous studies ranging from 11.5 to 23.2 cm^3^ [8–11, 15, 19, 20], and was greater in males than in females (*p* = 0.000). It was also greater on the right than left side (*p* = 0.046). Although the previous studies have reported that IOC types and angles may influence ION block or injury during surgery, their relationship to MSP remains unclear [2–7, 17, 18]. To the best of our knowledge, only one study examined the relationship between MS dimensions and IOC types [11], and one study evaluated the influence of MSV on IOC types and infraorbital ethmoid cells [15]. Osbon et al. [11] reported that the MS width and length (but not height) were positively correlated with increasing degree of the IOC protrusion. Kedar et al. [15] divided the MSV into three groups, as small (≤12 cm^3^), medium (>12 cm^3^ – <16 cm^3^), and large (≥16 cm^3^), and reported that the MSV was gender dependent, whereas there was no significant difference in terms of laterality. Similarly, we divided the sample into three groups according to MSV and found that the incidence of hypoplastic MS (34.6%) was higher than hyperplastic MS (27.1.6%), and males had more hyperplastic MS and IOCa than females (*p* = 0.025, *p* = 0.039). We suspected that the association between IOC types and IOCAs might be related to the MSP. Our results suggest that the lower IOCA1 and higher IOCA3 may be a product of hyperplastic MS, which mostly coexisted with Type III IOC. Correspondingly, Type III IOC had the lowest IOCA1 and the highest IOCA3 of the other IOCs. A notable finding in our study was an increase in MSV unit (1 cm^3^) adding 14.1% to the probability of having Type III IOC. The results of our investigation clearly indicate that hypoplastic MS was most commonly coexisted with Types I and IV IOCs, which did not protrude into the MS. Li et al. [17] reported that during extended medial maxillectomy approaches to the pterygopalatine fossa and infratemporal fossa, Type I IOC prevents ION identification in the orbital floor, which shows inferomedial positioning in hypoplastic MS, where mucosal thickening is common. Moreover, Types II and III IOCs can be used as a surgical landmark to identify the ION location as the surgeon dissects posteriorly [2, 13, 17].

On the other hand, logistic regression analysis results showed that older patients (especially females) were more likely to have hypoplastic MS, and the age seems to maintain a negative stronger association with the MSV compared to gender. Moreover, hyperplastic MS and larger IOCA3 were a significant predictive variables for the presence of Type III IOC followed by Type II IOC. Therefore, preoperative CT sections can be examined, taking into account that the likelihood of having Type III IOC was higher in adolescent male patients with hyperplastic MS. In contrast, older female patients were also more likely to have hypoplastic MS, in which the proportion of IOCa was lower. Thus, there may be huge differences in the localization of the IOF depending on whether the patient is female or elderly. Therefore, the presence of Types I and IV IOCs, especially in elderly patients with hypoplastic MS, may be another anatomical factor that increases the risk of insufficient regional block and precludes surgical safety and efficacy.

Limitations of this retrospective study include no assessment of IOF morphometry and MS mucosal thickening, no surgical data, single-centre healthy patient design, and a specific racial group, which limits its generalisability. Further studies investigating the relationship between IOC and MSV are needed with larger samples of different ethnicities and comparing unhealthy and control groups.

## Conclusion

To the best of our knowledge, this is the first study to describe the association between IOC types, IOCAs, IOCd, IOCa, and MSV. The results of this study suggest that older patients with hypoplastic MS are more likely to have Types I and IV IOCs, in which the IOF is mostly displaced laterally. Our results also revealed that the likelihood of having Types II and III IOCs, which are more prone to advanced protrusion and most commonly coexist with IOCd and IOCa, was higher in adolescent male patients with hyperplastic MS. Moreover, patients' age seemed to be the main factor contributing to MSV, followed by gender. Increasing the MSP may result in having larger IOCA3 and smaller IOCA1. It is important to clinicians to adequately know the anatomical variations of the MS, as the IOC is not the only canal traversing it. Therefore, the safety and success of surgical and anaesthetic procedures require a detailed knowledge of how MSV affects IOC morphology and angulation in relation to age and sex.

## Data Availability

The datasets generated during and/or analysed during this study are available from the corresponding author upon reasonable request.
